# Green Synthesis of Zwitterionic–Cyclodextrin Hybrid Polymer for Efficient Extraction of Polypeptides: Combination of Instrumental Analysis and DFT Calculation

**DOI:** 10.3390/polym17182524

**Published:** 2025-09-18

**Authors:** Xiaoyun Lei, Xin Wang, Yuzhe Cao, Bingxing Ren, Yanyan Peng, Hanghang Zhao

**Affiliations:** Shaanxi Key Laboratory of Catalysis, School of Chemical and Environmental Science, Shaanxi University of Technology, Hanzhong 723001, China; xiaoyunlei@snut.edu.cn (X.L.); 15829120426@163.com (X.W.); cyz2776960028@outlook.com (Y.C.); 13474695621@163.com (B.R.); peeiuyy@163.com (Y.P.)

**Keywords:** zwitterionic–cyclodextrin polymer, green analytical chemistry (GAC), in-tube solid-phase microextraction (IT-SPME), density functional theory (DFT) calculations

## Abstract

Adhering to the principles of green analytical chemistry (GAC) is crucial for advancing sample pretreatment. In this work, we developed a green in-tube solid-phase microextraction (IT-SPME) material utilizing non-toxic cyclodextrin and zwitterionic polymers as co-functioning monomers. The hybrid monolithic material was synthesized within 38 min via an efficient epoxy ring-opening reaction and free radical polymerization. Comprehensive characterization confirmed a rigid framework with strong anti-swelling properties, good permeability, and high enrichment efficiency on the polymers. When coupled with HPLC-UV, the optimized IT-SPME method enabled highly sensitive detection of polypeptides (vancomycin and teicoplanin) in aqueous matrices, achieving detection limits as low as 15.0–20.0 μg L^−1^, a wide linear range (60–800 μg L^−1^, R^2^ > 0.99), and good precision (RSDs = 5.9–8.2%). The prepared material demonstrated remarkable performance in real complex water samples, achieving recovery rates of up to 95.4%. Density functional theory (DFT) calculations indicated that the adsorption mechanism primarily involves hydrogen bonding and electrostatic interactions. This study presents an effective approach for the development of green chemical synthesis of extraction materials and offers a sustainable platform for monitoring trace contaminants in environmental waters.

## 1. Introduction

In-tube solid-phase microextraction (IT-SPME) has become an indispensable technique in the fields of environmental, pharmaceutical, biological, and food analyses due to its efficiency and versatility [1,2,3]. However, in the analysis of modern trace contaminants, it is crucial to strictly adhere to the principles of green analytical chemistry (GAC) throughout the entire IT-SPME workflow to minimize the environmental impact of the analytical process [4,5]. The core objective of the greening strategies in current IT-SPME research is to completely eliminate or significantly reduce the use of toxic volatile organic solvents and to substantially cut down the generation of hazardous waste. This trend is specifically reflected in several key developmental directions: the adoption of adsorbents based on renewable/bio-degradable materials (such as natural products), optimization of processes to reduce energy consumption and time, the development of simultaneous multi-target determination methods, the promotion of in situ measurement technologies, and the advancement of miniaturized and automated sample pretreatment and detection systems [6]. Notably, one method to uphold GAC principles is the incorporation of natural products and materials, such as chitosan, agarose, alginates, cellulose, starch, and cyclodextrins, as adsorbents or additives in IT-SPME technology for the preparation of adsorbent materials [7,8,9,10]. These materials typically exhibit high porosity, low mechanical resistance, and ease of chemical and/or physical surface modification, thereby expanding their applicability in the field of SPME.

Cyclodextrins (CDs) are cyclic oligosaccharides with a truncated cone structure, possessing a hydrophobic internal cavity and a hydrophilic exterior that enables selective host-guest encapsulation [11,12,13,14]. Owing to their high porosity, ease of modification, and molecular recognition capability, cyclodextrin-based polymers exhibit significant potential as efficient adsorbents or modification materials in in-tube solid-phase microextraction (IT-SPME) [15,16,17]. However, in practical applications, CD-based adsorbents indeed face several significant difficulties and challenges. For instance, the encapsulation of guest molecules by the cyclodextrin cavity primarily relies on size matching and hydrophobic interactions [18,19,20]. Although this provides affinity for specific-sized hydrophobic molecules, in complex real wastewater systems, various structurally similar or chemically similar pollutants may compete for adsorption sites. Additionally, naturally occurring organic matter, which is commonly present in water, may interact with cyclodextrins and occupy the cavity, thereby reducing adsorption efficiency [14]. Therefore, enhancing selectivity for specific target pollutants necessitates more meticulous molecular design (such as cyclodextrins modified with specific functional groups), significantly improving the specific adsorption capacity for target substances [21,22,23,24,25,26].

Meanwhile, the preparation of high-performance cyclodextrin adsorbents typically involves multi-step reactions, including the chemical modification of cyclodextrins, selection of appropriate carriers (nanomaterials, magnetic nanoparticles, polymeric materials, biomass carbon, MOFs, etc.), and surface activation [27,28,29,30,31]. For instance, synthesizing a magnetic molecularly imprinted β-cyclodextrin polymer for selective extraction requires over 72 h and involves multiple functionalization and polymerization steps [28]. Similarly, the development of an MOF-enhanced chitosan/β-cyclodextrin aerogel necessitates prolonged reaction times exceeding 48 h, high energy input, and labor-intensive purification processes [31]. Although these protocols improve adsorption performance, they generally demand precise control over immobilization conditions, extensive use of organic solvents, and complicated separation steps, which lead to high production costs and limited scalability. Furthermore, most hybrid adsorbents reported in recent studies still exhibit limited selectivity for specific targets and time-consuming preparation processes, often taking one to three days from initial modification to final column packing. Therefore, developing a rapid, efficient, and cost-effective method for preparing selectively modified cyclodextrins remains an essential and challenging goal in the field of SPME.

2-Methacryloyloxyethyl phosphorylcholine (MPC) is a representative zwitterionic polymer characterized by the presence of both cationic and anionic groups within the same monomer side chain, which results in an overall electrically neutral structure [32]. The strong electrostatic-induced hydration effect of the MPC head group imparts superhydrophilicity, enabling the formation of a dense hydrated layer that effectively resists nonspecific protein adsorption in biological environments, significantly minimizing interference from complex matrices [33,34]. Compared to materials such as polyethylene glycol (PEG), MPC demonstrates superior biocompatibility and stability, effectively addressing the rapid metabolic degradation often encountered with hydrophilic materials [35]. In the field of sample pretreatment, MPC can be copolymerized with nanoparticles (e.g., POSS) to create hybrid materials, leveraging its hydrophilicity, pH tolerance, and multiple action mechanisms (weak electrostatic, hydrophobic, anionic exchange) to efficiently enrich polar compounds in aqueous environments [36,37]. Consequently, to enhance the ability and selectivity of cyclodextrin (CD) materials in enriching polar targets in aqueous environments, it can be compounded with MPC [38]. This composite strategy integrates the superhydrophilicity and antifouling properties of MPC with the molecular recognition capabilities of cyclodextrin, resulting in a significant reduction of matrix effects while adhering to the principles of green microextraction, thereby improving detection accuracy.

This work developed a rapid, one-pot synthetic route utilizing an ionic liquid medium for the ultrafast preparation of a zwitterionic–cyclodextrin hybrid monolithic material in accordance with GAC strategies. Specifically, the glycidyl methacrylate-grafted hydroxypropyl-γ-cyclodextrin (GMA-HP-γ-CD) was synthesized as a functional monomer through a ring-opening reaction catalyzed within 30 min. This monomer was then copolymerized with 2-methacryloyloxyethyl phosphorylcholine (MPC) via a one-pot free radical polymerization reaction, resulting in the rapid formation of poly(GMA-HP-γ-CD-co-MPC) hybrid monolithic columns at elevated temperatures within 8 min. A series of characterization techniques, including scanning electron microscopy (SEM), Fourier transform infrared spectroscopy (FT-IR), energy-dispersive spectroscopy (EDS), thermogravimetric analysis (TGA), and water contact angle (WCA) measurements were adopted to characterize the properties of the materials. The parameters affecting the enrichment and elution processes, such as the composition of the enrichment and elution solutions and the sample loading flow rate, were optimized. The prepared material was then utilized as an IT-SPME adsorbent in conjunction with HPLC-UV methods to enrich and separate trace polypeptide residues in a real water environment. Density functional theory (DFT) calculations were conducted to verify the enrichment mechanism and explore potential underlying mechanisms.

## 2. Materials and Methods

### 2.1. Regents and Materials

Teicoplanin and vancomycin were obtained from Macklin Biochemical Technology Co., (Shanghai, China). Hydroxypropyl-γ-cyclodextrin (HP-γ-CD), 1,8-diazabicyclo[5.4.0]undec-7-ene (DBU), and n-hexanol were purchased from Shanghai Macklin Biochemical Technology Co., Ltd. (Shanghai, China). Methanol and formic acid were sourced from Tianjin Tianli Chemical Reagent Co., Ltd. (Tianjin, China). Glycidyl methacrylate (GMA) was acquired from TCI (Shanghai, China) Chemical Industry Co., Ltd. (Shanghai, China), while 2-methacryloyloxyethyl phosphorylcholine (MPC) was supplied by Shanghai Titan Scientific Co., Ltd. (Shanghai, China). Dimethyl sulfoxide (DMSO) was obtained from Shandong Keyuan Biochemical Co., Ltd. (Shandong, China). N,N′-Methylenebisacrylamide (MBA, 99%) was procured from Beijing Bailingwei Technology Co., Ltd. (Beijing, China). 1-Hexyl-3-methylimidazolium tetrafluoroborate ([C_6_mim][BF_4_]) was provided by Shanghai Chengjie Chemical Co., Ltd. (Shanghai, China). N,N-Dimethylformamide (DMF) was sourced from Tianjin OuBokai Chemical Co., Ltd. (Tianjin, China), and azobisisobutyronitrile (AIBN) was purchased from Shanghai Reagent Fourth Factory (Shanghai, China). All reagents were used as received without further purification.

### 2.2. Instrumentation

The surface topographical features and elemental constituents of polymers were investigated utilizing scanning electron microscopy (SEM) (Verios G4 UC, Thermo Scientific, Waltham, MA, USA). For pore structure evaluation, the zwitterionic monolithic material underwent Brunauer–Emmett–Teller (BET) surface area quantification and Barrett–Joyner–Halenda (BJH) pore size distribution analysis via nitrogen adsorption–desorption isotherms, measured on an ASAP 2460 system (Micromeritics Instrument Corp., Norcross, GA, USA). Chemical functional groups were identified through Fourier transform infrared spectroscopy (Thermo Fisher Nicolet iS50, Madison, WI, USA) with spectral acquisition spanning 400–4500 cm^−1^ at 4 cm^−1^ resolution with 32 scans. Hydrophilicity assessment employed dynamic water contact angle measurements (DSA25S, KRÜSS GmbH, Hamburg, Germany) using the sessile drop method. The surface elemental composition was analyzed using X-ray photoelectron spectroscopy (XPS) (ESCALAB 250, Thermo VG Scientific, Waltham, MA, USA) under ultra-high-vacuum conditions maintained at a pressure of less than 10^−8^ torr. Thermal decomposition behavior was probed by thermogravimetric analysis (PerkinElmer STA449C, Rodgau, Germany) under continuous N_2_ flow (50 mL/min), with programmed heating from ambient temperature to 800 °C at 30 °C/min.

### 2.3. Preparation of the Poly(GMA-HP-γ-CD-co-MPC) Column

**Figure 1:**
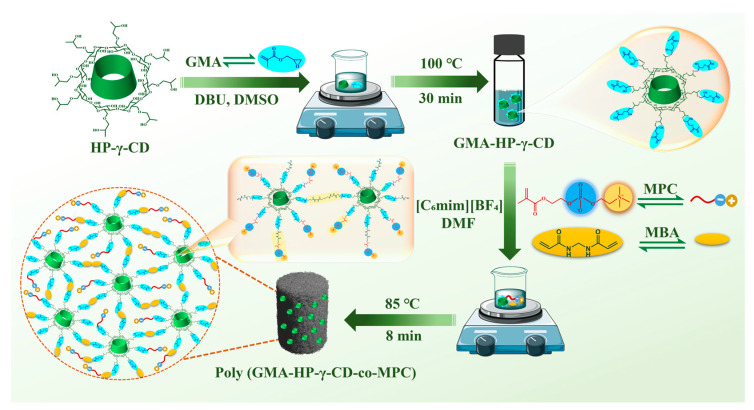
Schematic preparation of poly(GMA-HP-γ-CD-co-MPC) hybrid monolithic columns.

Before preparing the green cyclodextrin hybrid microextraction column, it is essential to pre-treat the inner wall of the silica capillary (530 μm I.D. × 690 μm O.D., Yongnian Optic Fiber Plant, Handan, China). This step aims to open the silicon–oxygen bonding structure on the inner wall, thereby exposing the silicon hydroxyl groups, which facilitate subsequent polymerization reactions with the hybrid materials [39]. As shown in Figure 1, accurately weigh 60 mg of HP-γ-CD, 16 mg of GMA, 6.0 mg of DBU, and 150 mg of anhydrous DMSO into a sample bottle, sealing it with a rubber septum screw cap. First, sonicate the mixture at 15 °C for 15 min, then heat it in a water bath at 100 °C for 30 min to prepare the GMA-HP-γ-CD monomer. After the polymerization solution cools to room temperature, take 5.0 mg of the synthesized monomer GMA-HP-γ-CD, 20 mg of MBA, 36 mg of [C_6_mim][BF_4_], 2.5 mg of MPC, 1.0 mg of AIBN, and 144 mg of DMF. Mix these components using vortex shaking to form a homogeneous solution. After sonicating the resulting solution at 15 °C for 15 min, slowly inject the polymerization mixture into the pre-treat capillary. Finally, seal both ends of the capillary with silicone plugs and immerse it in a pre-adjusted 85 °C water bath for 8 min. After the polymerization reaction is complete, thoroughly wash the polymer hybrid column with methanol to remove unreacted chemicals and gas pores. Finally, the prepared poly(GMA-HP-γ-CD-co-MPC) column could be cut to an effective length of 3.0 cm as needed for the following enrichment applications.

### 2.4. IT-SPME Procedure

The IT-SPME apparatus utilized in this study consisted of a 3.0 mL PEEK chromatography quantitative ring and a liquid chromatography pump (Series II, LabAlliance, State College, Centre County, PA, USA). Prior to each CME run, a 3.0 cm length of the column was cut and preconditioned with 1.0 mL of methanol at a flow rate of 60.0 μL min^−1^. A 3.0 mL sample solution, prepared by diluting the stock solutions in a loading solution consisting of 95% MeOH in water, was loaded onto the column at a constant flow rate of 60.0 μL min^−1^. Subsequently, a 1.0 mL methanol solution was used to elute interferences from the column at the same flow rate. The analytes were then eluted at 70.0 μL min^−1^ using a solution of MeOH/H_2_O (50:50, *v*/*v*) containing 0.2% formic acid. Finally, a 30 μL volume of the eluent was collected in centrifuge tubes and filtered for further HPLC-UV analysis. The blank control experiment was conducted following the same steps, excluding the target analyte.

### 2.5. HPLC-UV Condition

Chromatographic analysis was performed using a Shimadzu SPD-M20A HPLC system (Shimadzu Corporation, Kyoto, Japan), which was equipped with two LC-20AD solvent delivery units, a SIL-20A autosampler, an SPD-M20A diode array detector (DAD), and a CTO-20AC column oven. Separation was achieved on a Caprisil C18-X column (150 mm × 4.6 mm i.d., 3 µm particle size, 120 Å pore size). UV detection was set at a wavelength of 265 nm. The column temperature was maintained at 30 °C, and the injection volume was 5.0 µL. Mobile phase A consisted of 0.1% formic acid in water, while mobile phase B comprised 0.1% formic acid in methanol, delivered at a flow rate of 0.5 mL min^−1^. The analysis was conducted using the following gradient: initially, a 20% mobile phase B was maintained for 2.0 min, followed by an increase to 95% over 4.0 min, remaining constant until 13.0 min. After 19.0 min, mobile phase B was gradually reduced over a period of 3.0 min.

### 2.6. DFT Calculation

Density functional theory (DFT) calculations were used to investigate the molecular interactions between zwitterionic-modified polymers and polar peptide targets. All simulations were performed using the CP2K software package, version 9.1 [40]. Perdew–Burke–Ernzerhof (PBE) functional was employed in conjunction with the DZVP-MOLOPT-SR-GTH basis set [41]. To account for van der Waals interactions, the D3BJ dispersion correction scheme was incorporated [42]. Geometry optimizations were performed under these conditions to characterize the electronic and structural properties of the systems under investigation.

### 2.7. Sample Pretreatment

The prepared poly(GMA-HP-γ-CD-co-MPC) hybrid monolithic column was employed for the enrichment and detection of residual antibiotics, specifically vancomycin and teicoplanin, in environmental water samples collected from the Hanjiang River. The water samples exhibited a pH of 7.6 and a total organic carbon (TOC) content of 2.8 mg/L. Sample preparation procedure consisted of several steps: First, the water samples were pre-filtered through 0.22 µm membrane filters to remove particulate impurities. Next, 1.0 mL of each water sample (Hanjiang River water) was mixed with 3.0 mL of 90% (*v*/*v*) aqueous methanol, and the resulting mixture was subjected to ultrasonication for 15 min. Stock solutions of the peptides were prepared by dissolving 1.0 mg of lyophilized peptides in 1000 μL of water containing 0.1% formic acid, and these solutions were stored at −20 °C. All solutions were diluted to various concentrations as required for the IT-SPME experiment and analyzed in 3–5 technical replicates.

## 3. Results

### 3.1. Synthesis of GMA-HP-γ-CD-co-MPC Polymers

As illustrated in Figure 1, the synthetic route for the functionalized monolithic material consists of two main steps. First, the GMA-HP-γ-CD monomer, which bears vinyl groups, is synthesized through a nucleophilic ring-opening reaction between the hydroxyl groups of HP-γ-CD and the epoxy group of glycidyl methacrylate (GMA). This reaction is catalyzed by DBU under alkaline conditions. Subsequently, the obtained monomer is copolymerized with the zwitterionic monomer MPC in the presence of the crosslinker MBA via free radical polymerization, utilizing an ionic liquid ([C_6_mim][BF_4_]) and DMF as the porogens. The resulting hybrid zwitterionic cyclodextrin-based polymer is synthesized rapidly within 8 min and is employed as the stationary phase for in-tube solid-phase microextraction (IT-SPME) in subsequent extraction experiments.

### 3.2. Characterization of GMA-HP-γ-CD-co-MPC Hybrid Monoliths

**Figure 2:**
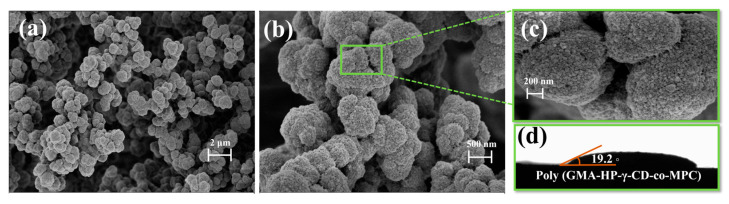
(**a**–**c**) SEM images of GMA-HP-γ-CD-co-MPC monolithic polymers. magnification: (**a**): 5000×; (**b**): 20,000×; (**c**): 50,000×; (**d**) water contact angle characterization.

As illustrated in Figure 2, the microstructure of the prepared hybrid monolithic columns was characterized using scanning electron microscopy (SEM). Figure 2a,b present the polymerization morphology of the monolithic columns at varying magnifications. It is evident that a uniform porous structure was established within the hybrid monolithic column material, characterized by uniformly distributed micropores and large through-holes in Figure 2c. The specific structure ensures high permeability and facilitates the extraction and enrichment of target substances. The contact angle of water on the surface of the hybrid monolithic material was characterized in situ using the sessile drop method, with the results depicted in Figure 2d. The contact angle of the prepared GMA-HP-γ-CD-co-MPC polymers was found to be 19.2°, indicating that this material possesses good hydrophilicity, thus providing a solid foundation for its specific enrichment detection of polar antibiotics in aquatic environments. Energy-dispersive spectroscopy (EDS) of the hybrid monolith (Figure S1) reveals the presence of elements C, N and P, confirming the incorporation of HP-γ-CD and the MPC monomer into the column matrix.

**Figure 3:**
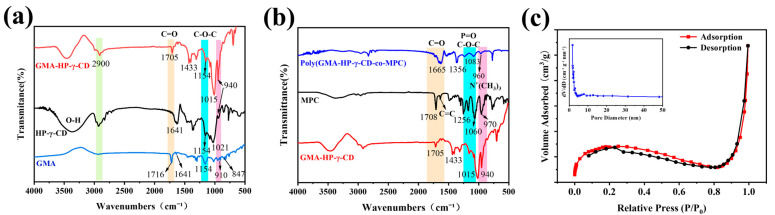
FT-IR spectra of (**a**) GMA, HP-γ-CD and GMA-HP-γ-CD; (**b**) GMA-HP-γ-CD, MPC and GMA-HP-γ-CD-MPC hybrid monolith; (**c**) N_2_-adsorption/desorption isotherms (inside: pore size distribution) of the poly(GMA-HP-γ-CD-MPC) monolithic column.

The polymerization of the overall column was characterized using FT-IR spectroscopy, as illustrated in Figure 3a. The peak at 910 cm^−1^ in glycidyl methacrylate (GMA) corresponds to the symmetric deformation vibration of the epoxy ring, while the peak at 847 cm^−1^ is attributed to the asymmetric deformation vibration of the epoxy ring. The peak at 1154 cm^−1^ is associated with the asymmetric stretching vibration of the ether bonds (C-O-C) present in the ester group of the GMA molecule. The peak at 1641 cm^−1^ corresponds to the stretching vibration of the carbon–carbon double bond (C=C), and the peak at 1716 cm^−1^ is linked to the stretching vibration of the carbonyl group (C=O) in the ester group. The characteristic skeletal vibration peak of HP-γ-CD’s cyclodextrin is found at 1021 cm^−1^ and 1154 cm^−1^, which correspond to the C-O stretching vibration and the asymmetric stretching vibration of the ether bonds (C-O-C) in the sugar ring, respectively. The peak observed at approximately 2900 cm^−1^ in both HP-γ-CD and the GMA-HP-γ-CD copolymer is attributed to the C-H stretching vibrations. These vibrations specifically arise from the symmetric and asymmetric stretches of the aliphatic methylene (-CH_2_-) and methyl (-CH_3_) groups present in the hydrocarbon skeletons of the cyclodextrin molecule, along with its hydroxypropyl substituents and the glycidyl methacrylate monomer. The bending vibration of H-O-H is observed at 1641 cm^−1^, and a broad and intense O-H stretching vibration peak is present in the range of 3100–3600 cm^−1^. In Figure 3b, the stretching vibration peaks at 1256 cm^−1^, 1060 cm^−1^, and 970 cm^−1^ in the MPC monomer correspond to the characteristic peaks of P=O, P-O-alkyl, and N+(CH_3_)_3_, respectively. Similar characteristic peaks are also observed in the polymer poly(GMA-HP-γ-CD-co-MPC), confirming the successful polymerization. BET images presented in Figure 3c proved that the hybrid monolithic columns demonstrate type IV isotherms, which are typical for mesoporous materials. The pore size distribution reveals the presence of a limited number of mesopores (2–15 nm), along with an average pore diameter of 10.59 nm, which together contribute to a specific surface area of 29.3215 m^2^/g.

**Figure 4:**
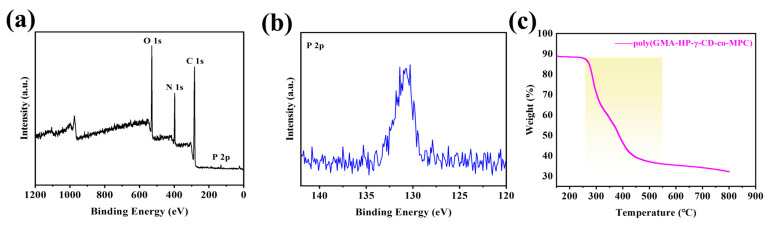
(**a**) The full survey of XPS spectrum; (**b**) the peak differentiation fitting of P 2p; (**c**) TGA curves of the poly(GMA-HP-γ-CD-MPC) monolithic column (Yellow region shows the primary decomposition stage with a sharp weight loss).

The surface elements, electronic, and chemical characteristics of the prepared hybrid materials were characterized using X-ray photoelectron spectroscopy (XPS), as illustrated in Figure 4a,b. The full-spectrum scan of the polymer material reveals peaks corresponding to O 1s, N 1s, C 1s, and P 2p, confirming the successful bonding of the γ-CD and MPC bifunctional monomers to the hybrid composite column. To assess the thermal stability of the prepared hybrid composite column, thermogravimetric analysis (TGA) was performed on the thermally polymerized hybrid column. As shown in Figure 4c, the synthesized polymer material begins to decompose at approximately 250 °C and exhibits complete decomposition around 520 °C. These results indicate that the synthesized GMA-HP-γ-CD-co-MPC hybrid material possesses sufficient thermal stability under realistic application conditions, confirming its suitability for IT-SPME in environmental monitoring scenarios.

### 3.3. Optimization the IT-SPME Conditions

#### 3.3.1. Influence of Sample Loading Solution

**Figure 5:**
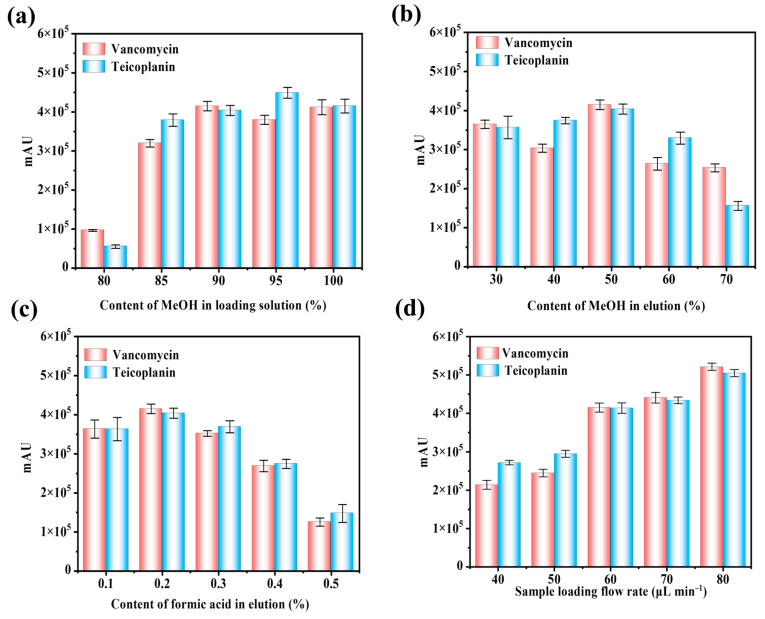
Influence of IT-SPME conditions on the extraction performance of vancomycin and teicoplanin on poly(GMA-HP-γ-CD-MPC) columns. The approach was evaluated from the measured HPLC-UV peak area after the extraction. (**a**) MeOH content in loading solution; (**b**) MeOH content in elution solvent; (**c**) Formic acid content of elution solvent; (**d**) flow rate of sample loading (*n* = 3).

In order to enhance the enrichment detection performance of the polymer material poly(GMA-HP-γ-CD-co-MPC) for vancomycin and teicoplanin, this experiment optimized parameters affecting PMME performance, including the composition of the enrichment solution, elution solution, pH, and sample loading flow rate. Peak area was used to evaluate extraction efficiency. Since the target compounds, vancomycin and teicoplanin, are polar compounds with good water solubility, the zwitterionic hybrid column can capture the targets through non-covalent interactions (such as electrostatic interactions, hydrogen bonding, hydrophilic interactions, and π-π interactions) between the stationary phase and the target compounds. A methanol/water mixture was used as the enrichment solution in this experiment, where the methanol content is a crucial factor affecting the extraction performance of the hybrid column. As shown in Figure 5a, as the methanol content in the enrichment solution increased from 80% to 100%, the enrichment efficiency of both target compounds increased and then slightly decreased. Therefore, 90% methanol–water was selected as the optimal enrichment solution for subsequent experiments.

#### 3.3.2. Influence of Composition and pH on Elution

The composition of the eluent plays a critical role in the desorption of analysis, considering the non-covalent interactions between the analytes and the IT-SPME stationary phase. In this experiment, a mixture of formic acid with water and methanol was selected as the elution, as illustrated in Figure 5b. As the ratio of formic acid–water to methanol increased from 30% to 70%, the extraction efficiency of the target analytes improved. However, the reduction in the content of polar solvents adversely affected the solubility of polar peptides. Consequently, a methanol/water mixture (50:50, *v*/*v*) containing 0.2% formic acid was ultimately determined to be the optimal elution ratio.

The electrostatic interactions between zwitterionic-modified cyclodextrin hybrid columns and analytes represent one of the primary extraction mechanisms. Therefore, the pH of the elution is a significant parameter that substantially influences the extraction performance of the hybrid column. To prevent peak splitting of the target analytes during HPLC-UV analysis, which may arise from the use of various acids, formic acid was consistently chosen as both the mobile phase additive and pH modifier in this study. As depicted in Figure 5c, the enrichment efficiency of the hybrid column for the two types of analytes initially increases and then slightly declines as the proportion of formic acid in the eluent rises from 0.1% to 0.5%. Although MPC is a zwitterionic compound, it retains a negative charge across a broad pH range, thereby facilitating cation exchange interactions. Thus, the observation could be attributed to the progressively enhanced cation exchange interactions between the target analytes and the stationary phase with increasing pH. Consequently, a 0.2% formic acid mixture in 50% water to methanol was selected as the optimal eluent for extracting the target analytes.

#### 3.3.3. Influence of Sample Loading Flow Rate

During the IT-SPME process, the sample solution flowing through the entire column results in an enrichment effect. Consequently, the sample loading flow rate may influence both the interaction time and the degree of interaction between the analytes and the stationary phase of the hybrid microextraction column. To investigate the effect of sample loading flow rate on extraction efficiency, flow rates ranging from 40 to 80 μL min^−1^ were examined, as illustrated in Figure 5d. As the sample loading flow rate increased, the peak area of the analytes gradually increased. This phenomenon may be attributed to the fact that while a higher flow rate can reduce analysis time, it may also result in insufficient contact between the target analytes and the stationary phase. Therefore, considering both enrichment efficiency and analysis time, a sample loading flow rate of 60 μL min^−1^ was selected for further studies.

### 3.4. Potential Adsorption Mechanism by DFT Calculation

To enhance the effectiveness of zwitterionic-modified GMA-HP-γ-CD-co-MPC polymers in the precise recognition of peptides, it is essential to gain a comprehensive understanding of the molecular interactions that take place between the polymers and polar peptide targets. This study utilizes an atomistic modeling approach rooted in density functional theory (DFT), offering an extensive exploration of the fundamental interactions that occur between zwitterionic-base polymers and specific peptide targets (teicoplanin and vancomycin).

**Figure 6:**
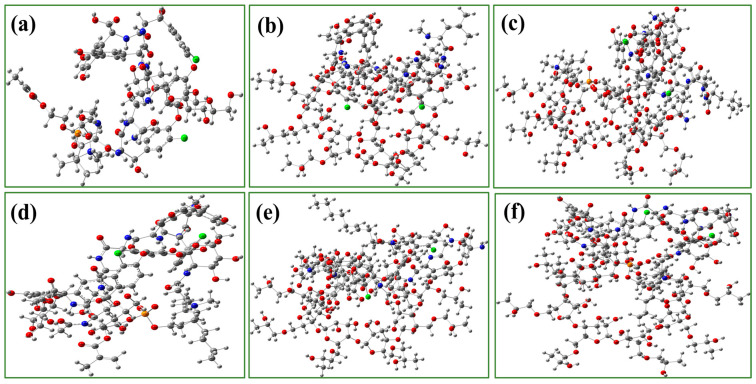
Optimal structure of a complex modeling the interaction of monomer/polymers with polypeptides (includes coordinate information). (**a**) MPC·vancomycin; (**b**) HP-γ-CD·vancomycin; (**c**) HP-γ-CD-MPC·vancomycin; (**d**) MPC·teicoplanin; (**e**) HP-γ-CD·teicoplanin; (**f**) HP-γ-CD-MPC·teicoplanin. Color code: carbon (gray), nitrogen (blue), oxygen (red) and phosphorus (orange).

Figure 6a–f present the optimized structures of complexes modeling interactions between monomers/polymers and polypeptide targets, with corresponding hydrogen bond positions, bond lengths and adsorption energy detailed in Table 1. The MPC·Vancomycin and MPC·Teicoplanin complexes exhibit relatively long hydrogen bonds (P–O···H), with bond lengths ranging from 6.04 to 11.86 Å. As illustrated in Figure 6a,d, the phosphate oxygen in the zwitterionic MPC monomer acts as a hydrogen bond acceptor, interacting with hydroxyl groups of the peptides. In comparison, complexes formed between HP-γ-CD and the same peptides show shorter hydrogen bonds, with lengths between 1.62 and 3.89 Å, facilitated primarily by hydroxyl groups on the glucose units (C2/C3) and hydroxypropyl moieties of HP-γ-CD acting as hydrogen bond donors (Figure 6b,e).

Notably, the hybrid HP-γ-CD–MPC polymer complex exhibits intermediate hydrogen bond lengths, spanning 2.32 to 3.90 Å for both P–O···H and O–H···O types. This reduction in bond length compared to pure MPC complexes indicates a strengthening of hydrogen bonding interactions, which could be attributed to the cooperative binding environment provided by the hybrid material. Moreover, the enhanced adsorption performance likely arises not only from optimized hydrogen bonding, but also from additional non-covalent interactions, such as electrostatic forces facilitated by the zwitterionic MPC moiety and hydrophobic effects contributed by the cyclodextrin cavity which collectively stabilize the complex through multi-functional synergy.

**Figure 7:**
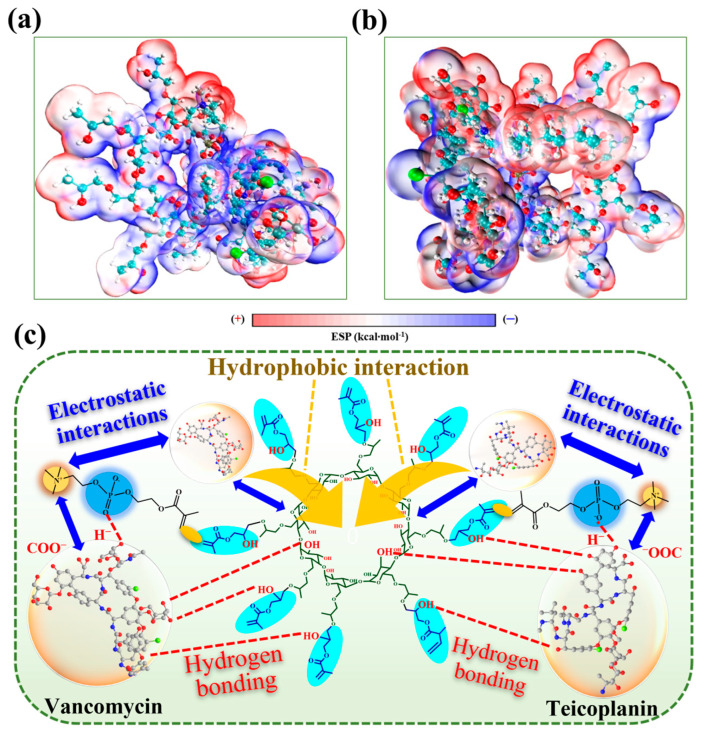
Electrostatic potential diagram of (**a**) GMA-HP-γ-CD-MPC·vancomycin; (**b**) GMA-HP-γ-CD-MPC·teicoplanin; (**c**) illustration depicting the potential interaction mechanism of zwitterionic-based hybrid polymers and polypeptide by DFT calculation (arrow denotes the hydrophobic interaction between cyclodextrin cavity and targets; dotted line represents the hydrogen bond).

**Figure 8:**
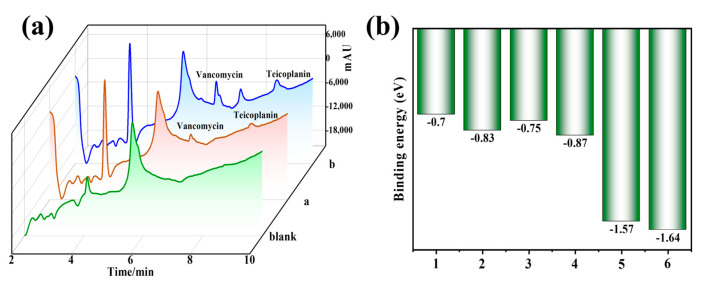
(**a**) The IT-SPME–HPLC-UV chromatograms of polypeptides in Hanjiang samples; blank: direct analysis of Hanjiang River samples; spiked with 70 μg L^−1^ analytes; (**b**) binding energies for different polypeptides on poly(GMA-HP-γ-CD-co-MPC) hybrid columns, 1: MPC·vancomycin, 2: MPC·teicoplanin, 3: HP-γ-CD·vancomycin, 4: HP-γ-CD·teicoplanin, 5: GMA-HP-γ-CD-MPC·vancomycin, 6: GMA-HP-γ-CD-MPC·teicoplanin.

The above conclusion is supported by results from electrostatic potential analysis. The zwitterionic MPC, consisting of negatively charged phosphate groups and positively charged quaternary ammonium salts, allows the HP-γ-CD-MPC hybrid polymer to exhibit significant electrostatic attraction towards the charged groups of target antibiotics. Generally, the electrostatic potential (ESP) mapping of the van der Waals (vdW) surface reveals regions characterized by high negativity (blue) and susceptibility (red) in terms of electrostatic potential. These regions indicate potential sites for electrophilic and nucleophilic attacks, respectively. As illustrated in Figure S2a,b, at physiological pH, the entire vancomycin molecule acquires a negative charge due to the ionization of the carboxyl group (pI ≈ 4.5), facilitating a robust binding interaction with the quaternary ammonium salt of MPC. Moreover, teicoplanin, which contains both carboxyl (negatively charged) and amino (positively charged) groups, establishes a bidirectional electrostatic attraction with MPC. Figure S2c,d illustrate that the hydroxyl groups of the hydroxypropyl substituents on HP-γ-CD may undergo slight ionization, resulting in a weakly negative charge that diminishes electrostatic interactions. Consequently, the hybrid polymer with zwitterionic modifications exhibits enhanced electrostatic interactions due to the significant electrostatic effects of MPC and the electrostatic assistance provided by HP-γ-CD, as shown in Figure 7a,b. In summary, the GMA-HP-γ-CD-MPC hybrid polymer developed in this study exhibits a remarkably strong adsorption capacity for vancomycin and teicoplanin, resulting from a multimodal synergistic mechanism that cooperatively integrates hydrogen bonding, electrostatic attraction, and hydrophobic interactions, as conceptually illustrated in Figure 7c. Ultimately, the highest binding energies achieved by the GMA-HP-γ-CD-MPC complex provides strong support for the proposed mechanism in Table 1 and Figure 8b.

### 3.5. IT-SPME-HPLC-UV Method Detection

#### 3.5.1. Method Validation

Under the optimized conditions for extraction, the assessment of the extraction efficiency of the designed poly(GMA-HP-γ-CD-MPC) hybrid monolithic column was conducted utilizing IT-SPME–HPLC-UV, with the outcomes presented in Table 2. The limits of detection (LODs), determined from signal-to-noise ratios (S/N = 3), were observed to be 15.0 and 20.0 μg L^−1^ for teicoplanin and vancomycin, respectively. The established linear ranges were 60–500 μg L^−1^ for teicoplanin and 80–800 μg L^−1^ for vancomycin. The run-to-run precision was recorded at RSDs of 5.9–8.2% (*n* = 3), respectively. These findings suggest that the IT-SPME–HPLC-UV method offers adequate sensitivity and reproducibility for analyzing polypeptides. Additionally, the poly(GMA-HP-γ-CD-co-MPC) column exhibited effective extraction capabilities even after 12 consecutive extraction processes.

#### 3.5.2. Real Sample Analysis

The poly(GMA-HP-γ-CD-co-MPC) hybrid column was successfully employed for the enrichment and detection of polypeptides in water samples collected from the Hanjiang River. As shown in Figure 8a, no targets were detected in the blank samples. To validate the method further, the real water samples were spiked with concentrations of 70, 150 and 300 μg L^−1^, and the recoveries ranged from 87.5% to 95.4% (see Table 3). The relative standard deviations (RSDs) were found to range from 4.8% to 9.2% by using the IT-SPME-HPLC-UV method. For the polypeptides adsorbed on poly(GMA-HP-γ-CD-co-MPC) hybrid monolithic columns, the binding energy (Eb) values are presented in Figure 8b. The highest binding energy of HP-γ-CD-MPC·vancomycin and HP-γ-CD-MPC·teicoplanin indicates that hydrogen bondings and electrostatic interactions between the columns and the target play significant roles in the extraction process. This finding is consistent with the DFT calculations, and all these results emphasize the accuracy and reliability of the current method in detecting polypeptide residues in real water samples.

### 3.6. Comparison with Other Reported Methods

As shown in Table 4, compared to conventional sorbents that require lengthy and multi-step preparation (4–24 h) [43,44,45,46], the proposed poly(GMA-HP-γ-CD-co-MPC) hybrid monolith was synthesized in just 38 min. Although its limit of detection (LOD) is higher than that of mass spectrometry (MS)-based methods, it provides sufficient sensitivity for polypeptide detection and excels in reducing organic solvent use, facilitating analysis automation and ensuring operational simplicity. This approach highlights an efficient and sustainable pathway for monitoring emerging organic contaminants in water.

## 4. Conclusions

This study presents an advancement in green analytical chemistry (GAC) through the design of a novel hybrid monolithic material for in-tube solid-phase microextraction (IT-SPME). The material is prepared by copolymerizing biodegradable and non-toxic cyclodextrin as a functional monomer with the zwitterionic compound 2-methacryloyloxyethyl phosphorylcholine (MPC), resulting in poly(GMA-HP-γ-CD-co-MPC). The obtained monolithic column combines the distinctive molecular recognition ability of cyclodextrin nanomaterials with the exceptional antifouling capacity and biocompatibility of zwitterionic polymers. Density functional theory (DFT) calculations indicate that the adsorption mechanism involves synergistic effects: MPC contributes strong specific ionic bonds, while HP-γ-CD provides hydrogen bonding, weak electrostatic interactions, and hydrophobic effects from its cavity. This cooperative behavior markedly enhances the adsorption capacity for target peptides such as vancomycin and teicoplanin, which is further corroborated by binding energy analysis and extraction performance. Compared to traditional solid-phase extraction (SPE) materials, the proposed monolithic column exhibits significant advantages, including cost efficiency, an eco-friendly synthesis process, and superior adsorption performance. This study underscores the successful integration of cyclodextrin-mediated molecular recognition and zwitterionic antifouling properties within a green synthesis paradigm, demonstrating considerable potential for future applications in monitoring diverse polypeptides in complex matrices such as industrial effluents.

## Figures and Tables

**Table 1:** Hydrogen bond position, bond length (Å) and adsorption energy (ΔE_ads_) at corresponding complex compounds.

Cluster	Hydrogen Bond Position *	Bond Length (Å)	Adsorption Energy ΔE_ads_ (kJ/mol)
MPC·vancomycin	P185-O187····H133	7.06	−67.54
	P185-O187····H121	9.13	
	P185-O187····H175	7.56	
	P185-O187····H124	11.86	
MPC·teicoplanin	P243-O245····H232	8.38	−80.08
	P243-O245····H152	6.04	
	P243-O245····H142	9.73	
	P243-O245····H141	10.66	
HP-γ-CD·vancomycin	O83-H189····O306	2.74	−72.36
	O270-H373····O11	2.89	
	O8-H136····Cl296	2.91	
	O84-H190····O324	3.89	
HP-γ-CD·teicoplanin	O95-H196····O304	1.95	−83.94
	O242-H31···O59	1.62	
	O242-H361····Cl133	3.12	
	O317-H423····O48	3.63	
HP-γ-CD-MPC·vancomycin	P9-O12····H197	3.90	−151.48
	P9-O12····H198	2.32	
	O95-H206····O20	3.61	
	O116-H224····O20	3.86	
HP-γ-CD-MPC·teicoplanin	P243-O245····H431	3.23	−158.24
	P243-O245····H151	2.62	
	O10-H145····O380	3.01	
	O25-H152····O254	3.77	

* The coordinate number information of atoms corresponds to Figure 6.

**Table 2:** Analytical performance for IT-SPME–HPLC-UV analysis of polypeptides.

Analytes	Regression Equation	Linear Range (μg L^−1^)	LOD (μg L^−1^)	LOQ (μg L^−1^)	RSD% (*n* = 3) Run-To-Run
Teicoplanin	Y = 318.14X − 15,005.7	60–500	15.0	50.0	5.9
Vancomycin	Y = 275.91X − 17,043.06	80–800	20.0	66.0	8.2

**Table 3:** Recoveries of the polypeptides from the Hanjiang River samples obtained by IT-SPME-HPLC-UV (*n* = 3).

Polypeptides	Added (μg L^−1^)	Recovery (%)	RSD (%)
Vancomycin	0	ND	-
	70	87.5	4.8
	150	91.5	5.1
	300	95.4	6.7
Teicoplanin	0	ND	-
	70	88.2	5.0
	150	93.8	6.7
	300	93.2	9.2

**Table 4:** Recent advances in solid-phase extraction for the analysis of antibiotics in water samples.

Analytical Methods	Sorbent	Antibiotics	Material Preparation Time	LOD (μg/L)	Linearity (μg/L)	Refs.
MSPE ^a^-HPLC-UV	Maltodextrin nanosponges	Fluoroquinolones	4–6 h	0.09–0.30	0.5–1000	[43]
MISPE ^b^-LC-MS/MS	Molecularly imprinted polymer	Fluoroquinolones	>24 h	0.002–0.007	0.01–50	[44]
MSPE-HPLC-UV	Chitosan-kaolin nanocomposite	Tetracycline	>24 h	0.09	0.3–100	[45]
SPE-HPLC-DAD ^c^	PVP/SWCNT-polyHIPE ^d^	Tetracycline	>12 h	0.07–0.30	0.5–100	[46]
SPME-HPLC-UV	GMA-HP-γ-CD-co-MPC polymer	Polypeptides	38 min	15.0–20.0	60–800	This work

^a^ Magnetic solid-phase extraction. ^b^ Molecularly imprinted solid-phase extraction. ^c^ Diode array detector. ^d^ Polyvinylpyrrolidone/single-walled carbon nanotubes incorporated polyhipe monolith.

## Data Availability

The original contributions presented in this study are included in the article. Further inquiries can be directed to the corresponding authors.

## Supplementary Materials

The following supporting information can be downloaded at: https://www.mdpi.com/article/10.3390/polym17182524/s1, Figure S1: Energy-dispersive spectrum (EDS) analysis of GMA-HP-γ-CD-co-MPC monolithic polymer; Figure S2: Electrostatic potential diagram of (a) MPC·vancomycin; (b) MPC·teicoplanin; (c) HP-γ-CD·vancomycin; (d) HP-γ-CD·teicoplanin.
