# Supplementary material for: Green Synthesis of Zwitterionic–Cyclodextrin Hybrid Polymer for Efficient Extraction of Polypeptides: Combination of Instrumental Analysis and DFT Calculation
Source: Polymers (Basel). 2025 Sep 18;17(18):2524. doi: 10.3390/polym17182524 (PMC12473841; doi:10.3390/polym17182524)
Supplement: Supplementary file 1 [file polymers-17-02524-s001.zip › polymers-3838441-Supplementary Materials.pdf]

Supplementary Materials

Green Synthesis of Zwitterionic-Cyclodextrin Hybrid Polymer for Efficient Extraction of Polypeptides: Combination of Instrumental Analysis and DFT Calculation

Xiaoyun Lei, Xin Wang, Yuzhe Cao, Bingxing Ren, Yanyan Peng, Hanghang Zhao*

* Shaanxi Key Laboratory of Catalysis, School of Chemical and Environmental Science, Shaanxi University of Technology, Hanzhong 723001, China

* Corresponding authors: E-mail: zhaohh931106@163.com

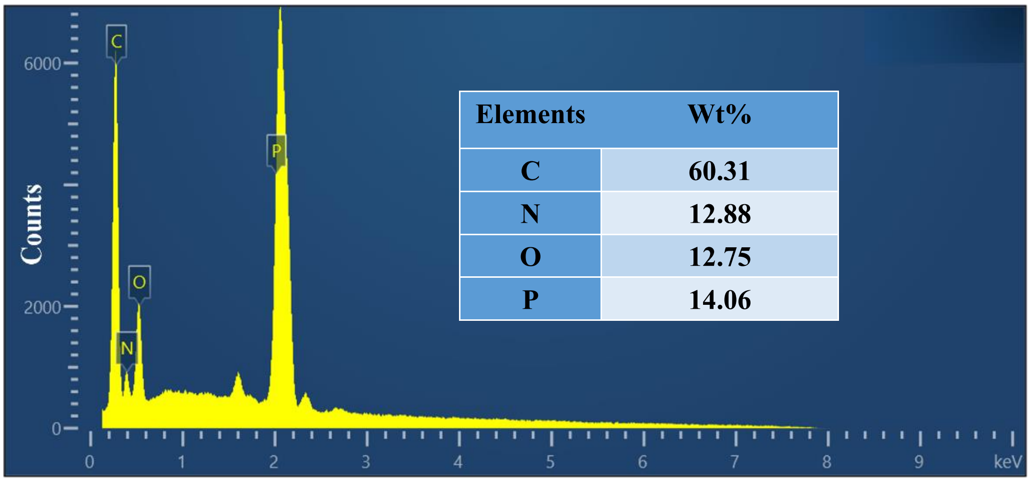

Figure S1. Energy dispersive spectrum (EDS) analysis of GMA-HP-γ-CD-co-MPC monolithic polymer.

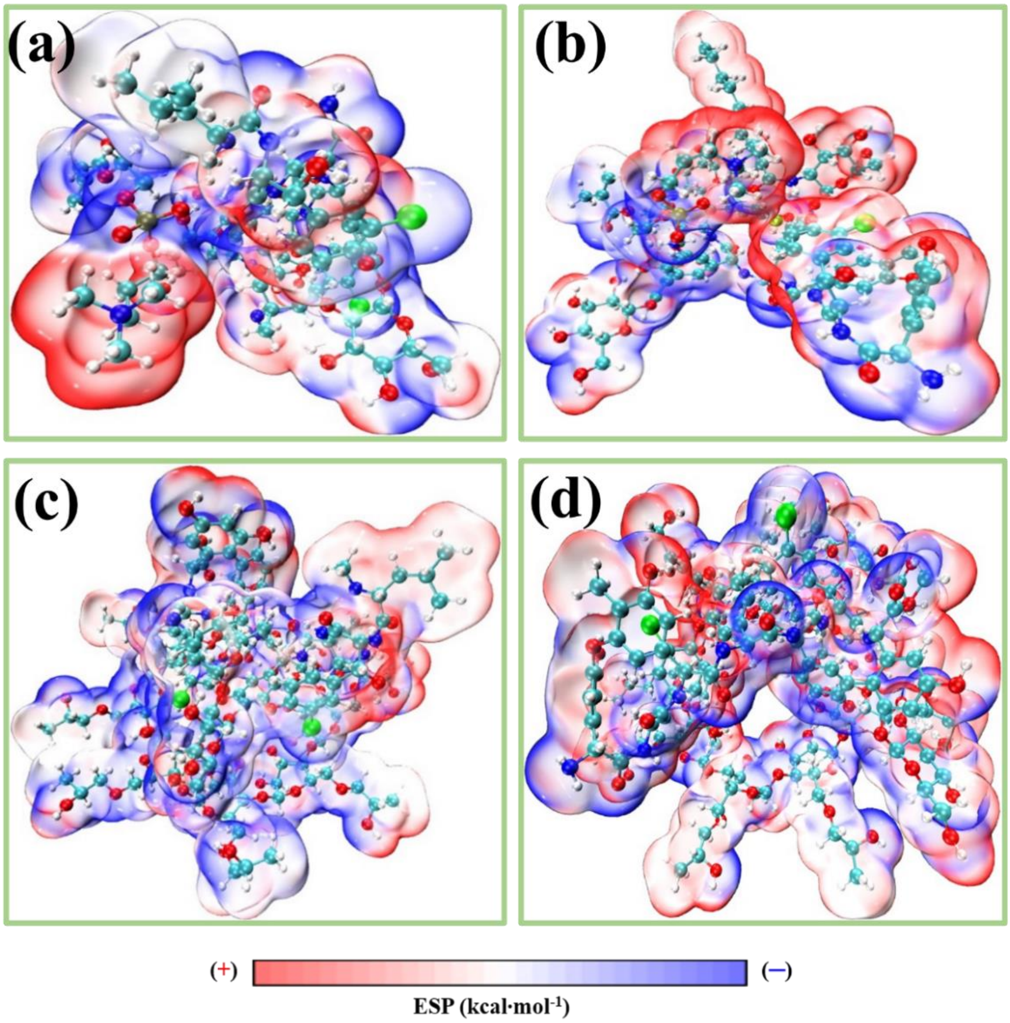

Figure S2. Electrostatic potential diagram of (a) MPC·vancomycin; (b) MPC·teicoplanin; (c) HP-γ-CD·vancomycin; (d) HP-γ-CD·teicoplanin.
